# Genomic and epidemiological analysis of *SARS-CoV-2* variants isolated in Guinea: a routine sequencing implementation

**DOI:** 10.1186/s12879-024-10411-2

**Published:** 2025-01-02

**Authors:** Aminata Mbaye, Haby Diallo, Thibaut Armel Cherif Gnimadi, Kadio Jean Jacques Olivier Kadio, Abdoul Karim Soumah, Joel Balle Koivogui, Jean Louis Monemou, Moriba Kowa Povogui, Djiba Kaba, Castro Hounmenou, Laetitia Serrano, Christelle Butel, Nicolas Fernandez Nuñez, Nicole Vidal, Emilande Guichet, Eric Delaporte, Ahidjo Ayouba, Martine Peeters, Abdoulaye Toure, Alpha Kabinet Keita, AFROSCREEN Team

**Affiliations:** 1 Centre de Recherche et de Formation en Infectiologie de Guinée (CERFIG), Université Gamal Abder Nasser de Conakry, Conakry, Guinea; 2 TransVIHMI, University of Montpellier, Institut de Recherche pour le Développement (IRD), INSERM, Montpellier, France

**Keywords:** Genomic, Epidemiology, *SARS-CoV-2*, Guinea

## Abstract

**Background:**

Several variants of *SARS-CoV-2* have a demonstrated impact on public health, including high and increased transmissibility, severity of infection, and immune escape. Therefore, this study aimed to determine the *SARS-CoV-2* lineages and better characterize the dynamics of the pandemic during the different waves in Guinea.

**Methods:**

Whole genome sequencing of 363 samples with PCR cycle threshold (Ct) values under thirty was undertaken between May 2020 and May 2023. The Illumina iSeq 100 technology was used. The sequences were then analyzed using the GeVarli pipeline to generate consensus sequences and variant calling. All sequences isolated in Guinea and available on GISAID were included in the analysis for phylogenetic tree and phylodynamic determination. Nextstain tools were used for these analyses. Statistical analysis was done using GraphPad Prism version 10.

**Results:**

The circulation of *SARS-CoV-2* in Guinea can be distributed in three different periods. The first, lasting from May to June 2020, was characterized by lineages B1 and B.1.1. The second period, from January 2021 to July 2021, was characterized by the lineages B.1.1.7 (Alpha), AY.122, B.1.1.318, R1, B.1.525 and B.1.629. The third period, between December 2021 and May 2023, was characterized by the Omicron variant, with nine subvariant majorities found. In addition, detecting variants in the period out of their circulation was documented. The importation and exportation investigation showed the strong movement viral association between Guinea and Senegal on the one hand and Guinea and Nigeria on the other.

**Conclusion:**

In summary, this study contributes to understanding the epidemic dynamics of the disease by describing the significant variants that circulated in Guinee and the distribution of this variant in the population. It also shows the importation and exportation of the virus during the pandemic. Sub-sampling and degradation of samples for sequences were observed. Organization and collaboration between laboratories are needed for a good sample-collecting and storage system for future direction.

**Supplementary Information:**

The online version contains supplementary material available at 10.1186/s12879-024-10411-2.

## Introduction

The severe acute respiratory syndrome coronavirus 2 (*SARS-CoV-2*) was first reported in December 2019 in Wuhan, China, and has rapidly spread to every country worldwide [1]. Globally, as of 05 December 2024, there have been 776,897,200 confirmed cases of COVID-19, including 7,076,329 deaths [2]. Epidemiological surveillance is one of the means to monitor the history of diseases, the dynamics of their development in time and space, and the different clinical forms that may exist. Genomic data can help to understand the evolution of viruses more rapidly. The changes occur as mutations or deletions are introduced in their genetic code.

For a virus like *SARS-CoV-2*, the emergence of variants over time is an expected phenomenon [3]. Several variants of *SARS-CoV-2* have demonstrated an impact on public health, including highly increased transmissibility, the severity of infection, or immune escape. They are identified through risk analyses and referred to as variants of concern (VOCs). The first variant of concern, he Alpha variant, B.1.1.7 or 20I/501Y.V1, was first detected in the United Kingdom in October 2020. It contains seventeen mutations, eight affecting the spike protein [4–7]. The Beta, B.1.351–20 H/501Y.V2 variants were discovered in South Africa as early as August 2020. Eight of its mutations also affect the spike protein, including the N501Y mutation and the E484K and K417N mutations. The Beta variant is distinguished by higher contagiousness (+ 25% compared to the initial strain), without being more lethal than the initial coronavirus [8–10]. The Gamma variant, or 20 J/501Y.V3, was subdivided into two subvariants, P1 and P2; they exhibit the E484K and N501Y mutations on the spike protein. This variant is highly contagious. It also affects a younger population than previous variants [10–12]. The Delta variant discovered in Central India in October 2020 quickly replaced the earlier variants, became responsible for an outbreak, and was classified as VOC. It has the particularity of being much more transmissible, hence its preponderance in the subsequent epidemic wave [13, 14]. The Omicron variant (21 K) is the latest VOC to emerge in late November 2021 in South Africa. Its diffusion rapidly increased, and the Omicron virus evolved rapidly in various variants [15–17]. An additional increase in its transmissibility has been observed [18].

Thus, in response to the regular emergence and worldwide diffusion of new variants, screening and sequencing methods were rapidly developed for the early detection and monitoring of *SARS-CoV-2* variants. Screening methods aim to identify variant-specific mutations on known variants within a maximum of 24 h after the diagnosis of infection. At the same time, sequencing remains the best method for confirming suspected variants.

For some African countries, genomic surveillance has been at the heart of the response from the start of the epidemic. It has been essential in tracking cases and sharing genomic data from the first imported cases [16, 19]. The first sequences reported by Nigeria [20], Uganda [21], and South Africa [22] made it possible to quickly identify the variants circulating on the continent and their transmission power. However, this was only the case for some African countries. Sequencing platforms were generally installed and functional in most African public health laboratories by the end of 2021 [23, 24]. The question was how to implement sequencing capabilities and set up a surveillance strategy in these countries.

The Republic of Guinea, located in West Africa with an area of 245,857 km2, is bordered by the Atlantic Ocean and six countries: Guinea Bissau in the north-west, Senegal in the north, Mali in the north-east, Côte d’Ivoire in the east, and Liberia and Sierra Leone in the south.

According to the WHO report on December 05, 2023, the number of estimated COVID-19 cases was 38,582, with 468 deaths. The management strategy to control the COVID-19 epidemic relied primarily on precise diagnosis by reverse transcriptase quantitative Polymerase chain reaction (qPCR), disease management by the hospitalization of cases, vaccination, adherence to barrier measures in the population, such as mask-wearing, use of hydro-alcoholic gel, physical distancing, and limitation of the number of passengers in standard transports, entry control with vaccination card at airport and other entry points (where body temperature of travelers was systematically checked). COVID-19 vaccination coverage in the country was around 36% for the population aged 12 years and above and 47% among people over sixty by April 20th, 2023 [25]. The study of *Diallo et al.* [25]. found that 75% of thegeneral population in Conakry, the capital city, had antibodies to *SARS-CoV-2*. Guinea was one of the countries where genomic surveillance of *SARS-CoV-2* was challenging for local laboratories.

This study aimed to better describe the distribution of *SARS-CoV-2* lineages during the different COVID-19 waves to characterize the dynamics of *SARS-CoV-2* variants in Guinea. The use of the genomic approach for *SARS-CoV-2* evolution characterization and its routine involvement is thus demonstrated here.

## Methods

### Study sites and characteristics of participants

Participants attended two COVID-19 treatment centers (CT-Epis Gbessia and Kenien), the municipal medical center of Matam, or the “Centre de recherche et de formation en infectiologie de Guinée” (CERFIG) to perform an RT-PCR test for diagnosis of COVID-19 infection. Whereas participants were symptomatic in the treatment and medical centers, those attending the CERFIG site needed a COVID-19 assessment for international travel. Many participants were from Conakry, the capital city, or Kindia, a neighboring region of Conakry. A total of 24,638 samples were assessed between May 2020 and May 2023. After acceptance, age, sex, collection date, contact with COVID-19 patients, and region of origin were collected as sociodemographic information.

### Sample collection and selection for sequencing

Nasopharyngeal swabs were collected and stored in viral transport media (VTM) tubes. *SARS-CoV-2* diagnosis was made by qPCR with the RunMei kit (Human Runmei Gene Technology Co., LTD, China). All positive samples for *SARS-CoV-2* with a cycle threshold (Ct) below 30 were selected for sequencing. If the patient had more than one test (test 1 and control), the sample from test 1 was chosen if the Ct was < 30 cycles.

### Library preparation and sequencing

Sequencing was done on extracted samples using the Runmei kit. From May 2020 to May 2022, libraries were prepared using the Illumina DNA prep protocol (Illumina Inc, USA). From June 2022, the COVIDSeq protocol from Illumina was used. Shortly after reverse transcription with random hexamers, tiled multiplex PCR was performed using *SARS-CoV-2* specific primers (Pool 1 & 2) in two separate reactions. The two reaction products were pooled per patient, cleaned, and quantified using the Qubit 3.0 fluorometer (Invitrogen Inc.). The amplified products were processed for Tagmentation and adapter ligation using Illumina set indexes. Clean-ups were conducted according to the manufacturer’s protocol. Samples were pooled in twelve batches to form the final library and quantified using the Qubit 3.0 fluorometer (Invitrogen Inc.). The final library was normalized to a concentration of 4nM. Then, a new dilution was conducted to obtain a final concentration of 75pM, as advised to be loaded onto the iSeq 100 platform. One or two negative controls were introduced in each library preparation.

### Genome assembly and *SARS-CoV-2* lineage assignment

We used the GeVarLi pipeline (GEnome assembly, VARiants calling, and Lineages assignment), a homemade pipeline developed for the AFROSCREEN sequencing network (https://forge.ird.fr/transvihmi/nfernandez/GeVarLi) for quality control, alignment, variant calling, mapping to reference genome and consensus sequence building. Low-quality sequences identified by NextClade (https://clades.nextstrain.org/) and in the GeVarLi report were excluded for phylogenetic construction.

To perform the phylogenetic analysis, we retrieved *SARS-CoV-2* sequences from Guinea from GISAID on September 10th, 2023 (Total sequences = 1032, including 260 sequences from this study). We removed sequences with no metadata and sequences that remained unassigned. We then separated non-Omicron and Omicron strains. These are because of the genetic distinctiveness of Omicron variants compared to the other variants and the transmission dynamics: Omicron variants are known for their high transmissibility [26, 27]. Each data set was aligned with Nextalign CLI v2.14.0. The first and last one hundred bases were masked relative to the reference strain sequence Wuhan-Hu-1 (Accession Number MN908947.3) to avoid ambiguities through primer contamination. Maximum likelihood trees for each alignment were inferred in IQ-TREE version 2.1.4, using fast model selection to measure confidence in phylogenetic tree branches. Non-Omicron and Omicron trees were inferred with a general time reversible (GTR) model of nucleotide substitution, using a proportion of invariable site (+ I) and a discrete Gamma model with default four rate categories. The number of bootstrap replicates was set to one thousand.

We produced a time-scaled phylogenetic tree based on sampling dates. Knowing that nucleotide changes among Omicron variants resulted in sixty-one amino acid changes. In comparison, the nucleotide changes in other VOCs showed eleven amino acid changes [28] using a rate of 8.0 × 10^−4^ nucleotide substitutions per site per year for non-Omicron and 6.0 × 10^−4^ for Omicron datasets in Tree Time v0.11.4. Before building the final tree, outliers that deviated more than three interquartile ranges from the root-to-trip regression were removed. Phylogenetic trees were visualized and annotated using Figtree v1.4.4.

To study the contact tracing of *SARS-CoV-2* strains isolated in Guinea, the software Microbe Trace (https://microbetrace.cdc.gov/) was used. For importation and exportation determination of the *SARS-CoV-2*, the pipeline Augur version 23.1.1 of Nextstrain (https://docs.nextstrain.org/projects/augur/en/stable/) was used. The input file was sequenced with good coverage available on GISAID from Guinea, Senegal, Sierra Leonne, Nigeria, Mali, Gambia, and Côte d’Ivoire, as well as the custom selection on GISAID. Auspice (https://docs.nextstrain.org/projects/auspice/en/stable/) was used to visualize the graph after analysis. QGIS v.3.32.1 was used to annotate Guinea’s geographical card. Other graphs and statistical analysis were realized using Graph-pad Prism v.10.4.0. the application Inkscape v.1.3 was used to refine the annotations.

## Results

### Description of samples processing to submission in the GISAID database

**Fig. 1:**
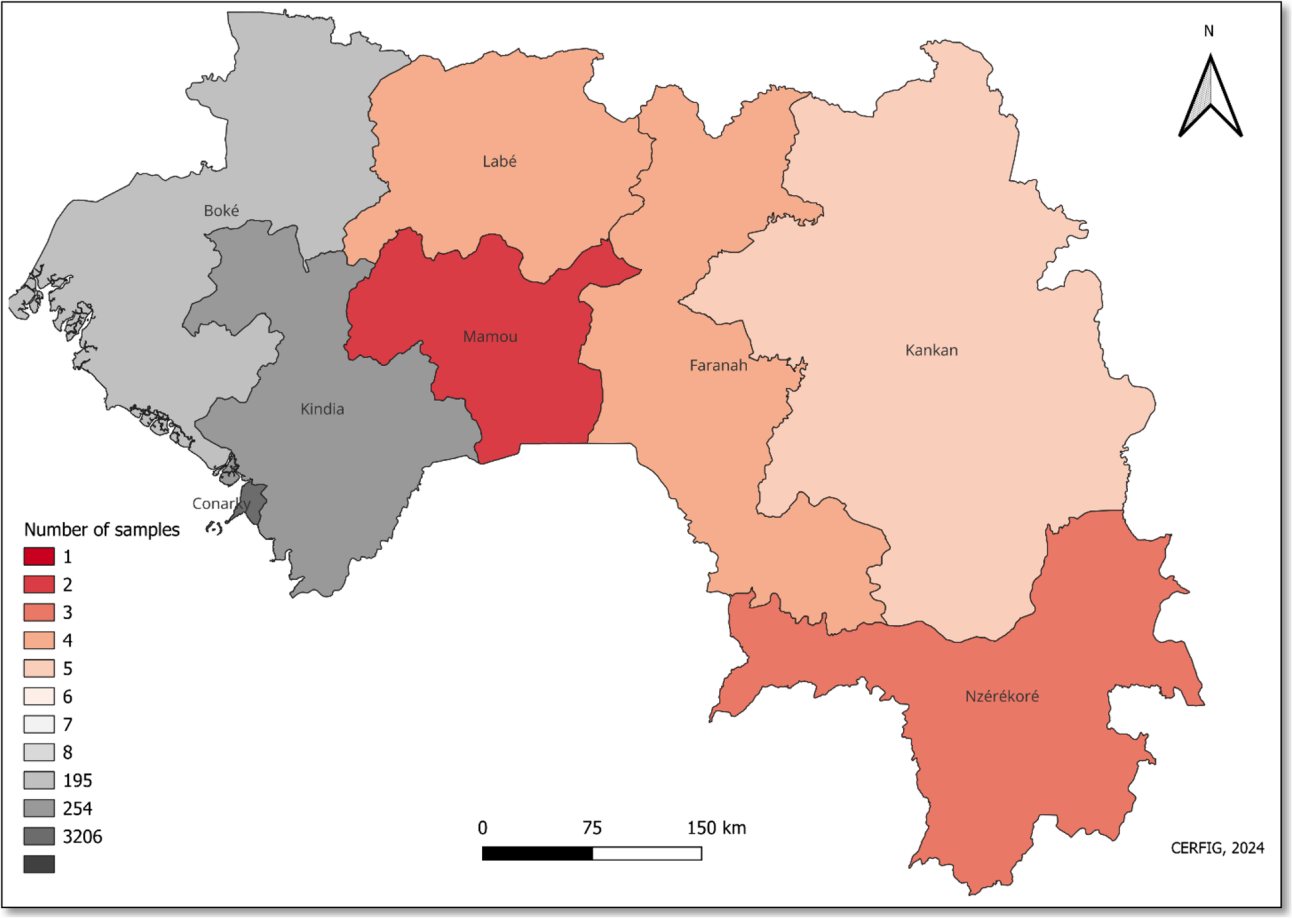
The number of samples collected by regions in Guinea In this study, the samples were isolated from 8 regions with a concentration in Conakry and their neighborhood regions Boke and Kindia (in grey). The coloration by region is dependent on the number of samples. Guinean administrative areas are the number 8 (Conakry, Kindia, Labe, Mamou, Boke, Kankan, Faranah, and N’Zérékoré)

Among 24,188 samples received in the CERFIG laboratory, 5697 were positive, and 655 samples displayed a Ct value under thirty. The genome sequencing was undertaken for 385 samples collected from May 2020 to May 2023. Retrospective analyses were done in 2022 for nineteen samples collected in 2021. A total of 299 samples could have their lineage assigned. 87% (260/299) of these samples were sequenced with a coverage of 30x > 69% and were thus submitted and accepted by the GISAID platform (Table 1). These samples came from all Guinean administrative regions, with 82,5% originating from the Conakry region (Fig. 1). Most (83%) sample genome sequences were covered between 95% and 100%. As already observed, the genome sequence coverage increased inversely relative to a lower Ct value (Table 2).

**Table 1:** Samples flux from collection to submission on GISAID

Samples	2020	2021	2022	2023	Total
Received	13,656	19,751	4653	898	38,158
Positives	3046	5598	871	102	9617
Negatives	10,371	13,980	3714	790	28,855
Good for sequencing (Ct < 30)	173	220	195	67	655
Sequenced	6	66	214	99	385
Deposited on GISAID	6	66	143	45	260

**Table 2:** Genome coverage (30X) and ct values relationship

Coverage 30X (%)	Number	Percentage (%)	Mean Ct (confident interval)
[69–80]	3	1%	26.80 (22.88–30.31)
[80–90]	12	5%	25.80 (21.78–29.87)
[90–95[	29	11%	23.51 (21.55–24.47)
[95–100]	216	83%	22.76 (21.08–22.44)
Total	260	100%	-

30x = sequencing depth

###  Socio-demographic and clinical characteristics of study participants and epidemic dynamics into distinct groups

**Fig. 2:**
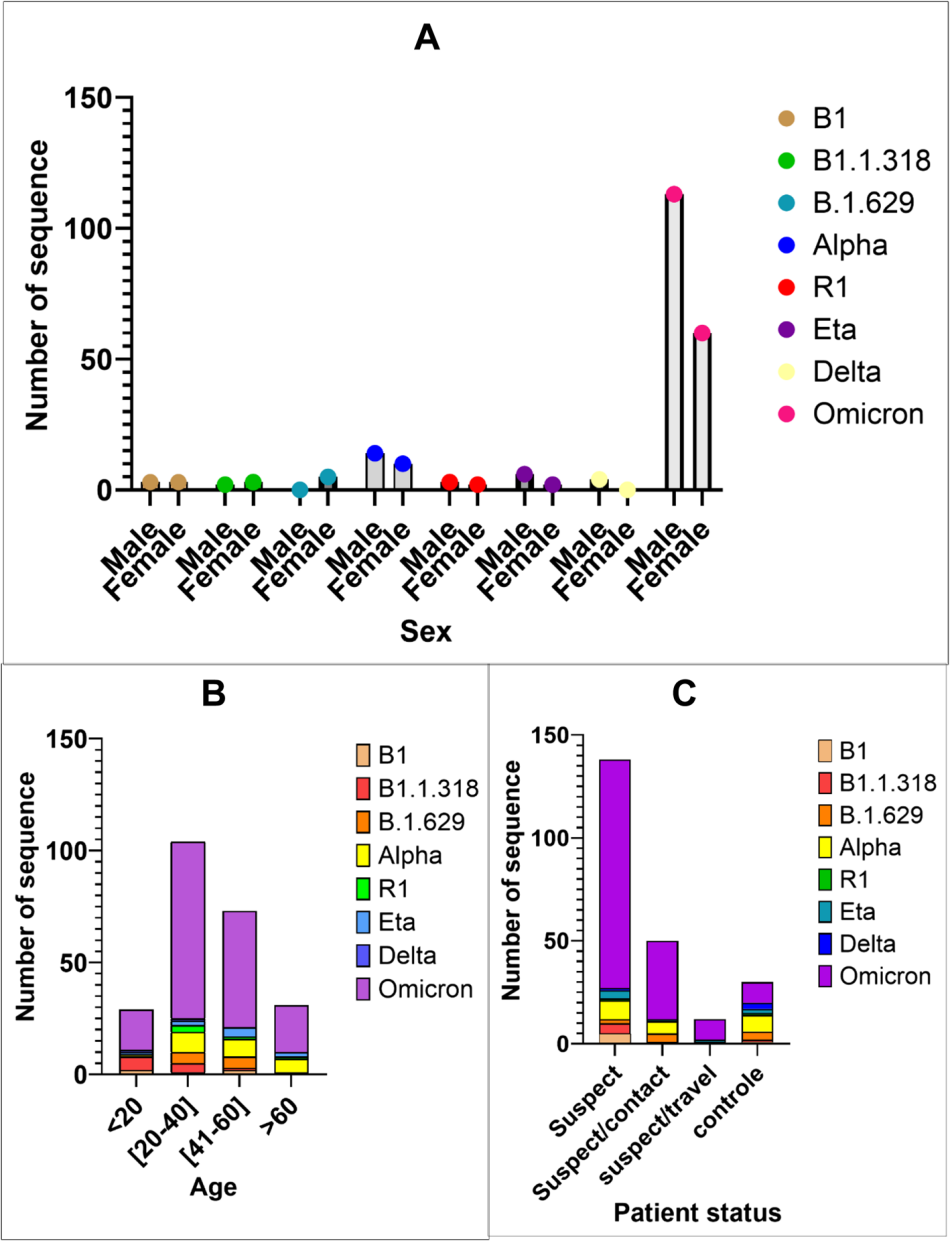
Distribution of sequences depending on **A**, the sex; B**,** the age; and **C**, the patient status

Eighty-four percent of samples were collected from suspects living in Conakry (Fig. 1). For Covid 19 positives by PCR participants, the male gender was represented at 64%, and 51% were between 20 and 40 years old. Followed by the persons aged between 41 and 60 (28.8%). The patient status was represented by control cases (62%), suspect cases (28%), suspect/contact cases (7.4%), and suspect/traveler cases (2.6%), primarily detected in the airport (Table 3). These results on exposition by sex were also observed in Fig. 2A, and we noted that the more we get to sample, the better we characterize this observation. According to Fig. 2B, only Delta and Omicron were detected for suspects/travelers. For Omicron (Fig. 2C), the observation is that although it was the most detected variant, the number of positive control patients was less than in the other categories.

**Table 3:** Socio-demographic and clinical characteristics of study participants

Description	Designation	Number (*n*)	Percentage (%)
Patient status	Control	5939	62
	Suspect	2667	28
	Suspect/Contact	729	7.4
	Suspect/Travel	265	2.6
	**Sub-total**	**9600**	**100**
Sex	Female	1294(3582)	35
	Male	2361 (5978)	64
	NA	23	1
	**Sub-total**	**3678**	**100**
Age (year)	< 20	340	9.1
	[20–40]	1928	51.9
	[41–60]	1070	28.8
	> 60	367	10
	NA	11	0.2
	**Sub-total**	**3716**	**100**

Control: Designate the patient positive for COVID-19 and do the control test to see their statute. One person can have until tree control. By calculating the distribution of Samples by age and sex, this category was eliminated. Suspects are patients who have COVID-19 clinical symptoms and do not know where they keep it. Contact: designate a person who had contact with a COVID-19 patient. Travel: designate a person who does the test for traveling, entering, or going out of the country

### Genomic epidemiology of *SARS-CoV-2* strains that circulated in Guinea

**Fig. 3:**
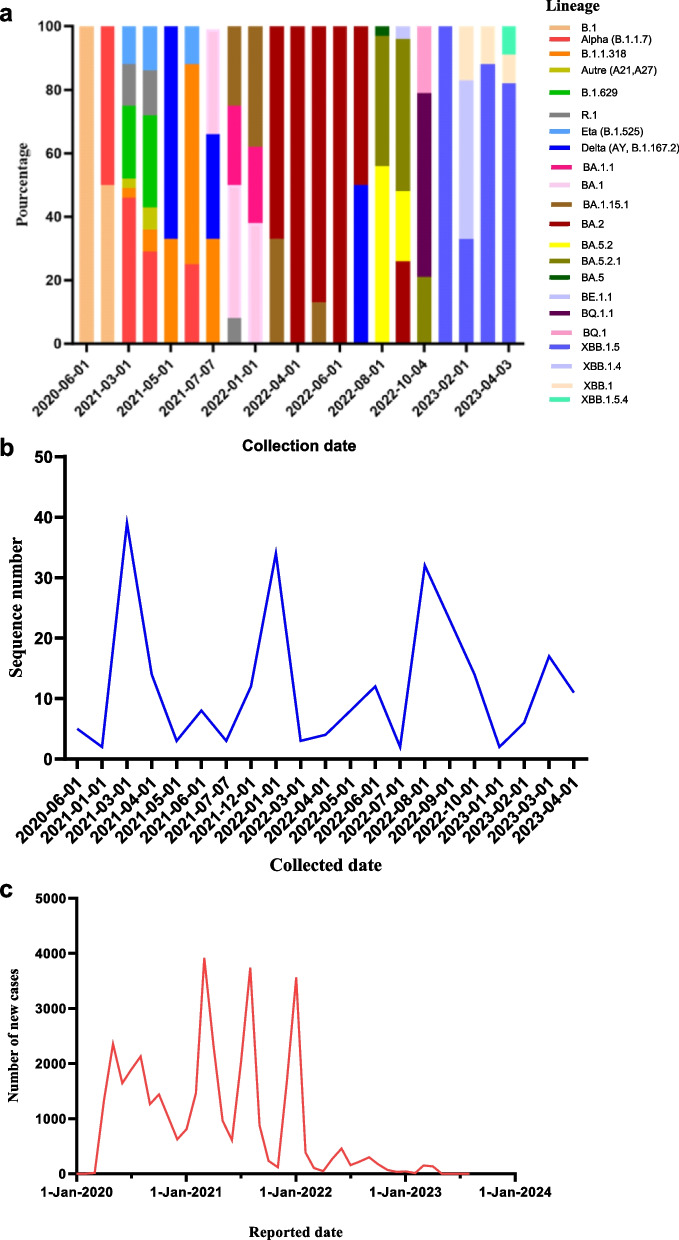
Representation of cases and *SARS-CoV-2* lineages detected in the time. **a** represents the variants found for each period. **b** represents the number of sequences generated by month in the laboratory. **c** indicates the number of cases of COVID-19 by day for Guinea, which is available at https://covid19.who.int/data. The correlation between the log of generated sequences and the number of new cases is positive and equal to 0.33. Only lineages mainly detected were reported in this graphic

**Fig. 4:**
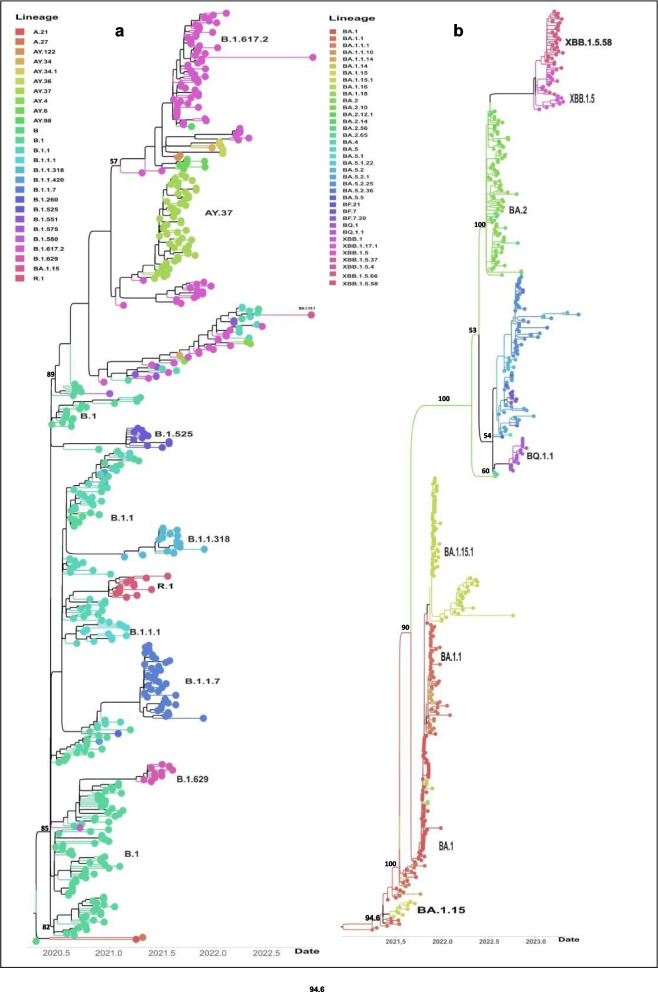
Phylogenetic and contact trace analysis of *SARS-CoV-2* strains isolated from Guinea. **a** indicate phylogenetic analysis of non-omicron strains, **b** indicate phylogenetic analysis of omicron strains, and date corresponds to the samples collected to date. The numbers on the branch designated the bootstrap support % values for nodes

Figure 3 shows the distribution of lineages over time (3a) for sequences isolated at CERFIG lab, the distribution of number of confirmed cases over time (3c) available on https://covid19.who.int/data and the number of sequences included in the study over time (3b). The phylogenetic analyses of all *SARS-CoV-2* strains sequenced from Guinea by different laboratories (CERFIG, Institut Pasteur de Guinee (IPG), Institut Nationale de Sante publique (INSP) and Laboratoire des Fièvres Hémorragique Virales en Guinée (LFHVG)) is available on GISAID shown in Fig. 4. Two trees were made, one for non-Omicron strains (Fig. 4a) and another for Omicron stains (Fig. 4b). From this figure and according to Table 4, the CERFIG laboratory sequenced more in the Omicron period between 2022 and 2023 than in the non-omicron period between 2020 and early 2022, with 73 and 176 sequences, respectively. Globally, the same lineages were detected for all laboratories, and this depending on the sampling period. Principally, lineages that circulated in Guinea (Table 4) were B.1(64), B.1.1 (13), B.1.1.1 (11), R.1 (15), B.1.1.318 (13), B.1.629 (18), B.1.1.7 (56), B.1.617.2 (46), AY.37 (42) and B.1.525 (15) for non-Omicron and Omicron lineages BA.1 (64), BA.1.1 (42), BA.1.1.1 (5), BA.5.2.1 (18), BF.1 (3), BA.5.2.25 (28), BQ.1 (3), BQ.1.1 (12), XBB.1.17.1 (7), XBB.1.5 (9) and XBB.1.5.58 (58). For these, the CERFIG lab isolated half of the samples for each lineage sequenced in Guinea, except for the first circulated lineages (B.1, B.1.1 and B.1.1.1) and Delta variant (B.1.617.2 and AY.37). According to the number of cases officially registered for the country (Fig. 3c), four main transmission periods from March 2020 to March 2022 and some slight peaks, at the end of the fourth period, were observed. The first period, starting in March 2020, was characterized by the presence of three sub-phases in May 2020 (cases number (n) = 2355), August 2020 (*n* = 2120), and October 2020 (*n* = 1438). Only variant B.1 collected in June 2020 was detected in six sequences for CERFIG. However, this first period corresponded to the circulation of lineages B.1 and B.1.1, as shown in Fig. 3a. The second period (Fig. 3c) lasted from December 2020 (*n* = 625) to June 2021 (*n* = 610), with a peak in March (*n* = 3916). In this period, the CERFIG laboratory (Fig. 3b) sequenced more in March (*n* = 39) and in April (*n* = 14). We noted the detection of Alpha (B.1.1.7), B.1.318, Eta (B.1.525), B.1.629, R1, B.1.1, and B.1 with a trend for Alpha predominance in March (Fig. 4A). Between May and June 2021, variants B.1.1.318, Alpha (B.1.1.7), B.1.1.318, and Eta in June 2021 cocirculated. Early detection of the Delta variant (Fig. 4A) was observed in May from two travelers, and the lineage isolated was AY.122. The same trend was observed in Guinea (Fig. 4a).

**Table 4:** Repartition of most frequent lineages isolated in Guinea in function of laboratories

Clade-WHO	Lineage	CERFIG	Others lab	Total
Other	B.1	6	132	138
	B.1.1	0	60	60
	B.1.1.1	0	11	11
	R.1(B.1.1.316.1)	8	9	17
	B.1.1.318	8	10	18
	B.1.629	13	8	21
Alpha	B.1.1.7	25	33	58
Delta	B.1.617.2	4	93	97
	AY.37	0	55	55
Eta	B.1.525	9	7	16
Subtotal non-Omicron.		**73**	**418**	**491**
Omicron	BA.1	20	54	74
	BA.1.1	15	44	59
	BA.1.1.1	1	6	7
	BA.1.15.1	20	60	80
	BA.2	28	53	81
	BA.5.2	4	6	10
	BA.5.2.1	18	6	24
	BF.7	3	5	8
	BA.5.2.25	18	18	36
	BQ.1	1	3	4
	BQ.1.1	9	7	16
	XBB.1.17.1	5	4	9
	XBB.1.5	8	4	12
	XBB.1.5.58	26	6	32
Subtotal Omicron		**176**	**276**	**452**
Total		**249**	**694**	**943**

Lineages identified with only one ice are not represented in these tables

The period between July 2021 and November 2021, according to Fig. 3a, was characterized by the large circulation of Delta variant with lineages B.1.617.2 and AY.37. Some sequences (*n* = 2) within R1 lineage and (*n* = 1) for Eta were also noted. The fourth period started in December 2021 (*n* = 1678), with a peak in January 2022 (*n* = 3565), and ended in April 2022 (*n* = 47). Also, we noted the detection of the R.1 lineage in December 2021. In early January 2022, the cocirculation of Delta, BA.1.15, and BA.1 detected by other laboratories was noted (Fig. 2b). The Omicron variant was the only one detected at the end of January. So, the cocirculation of BA.1, BA.1.1, and BA.1.15.1 from February to April 2022 was noted. A predominance of BA.2 was observed by the end of April 2022 (Figs. 3a and 4b). The end of this period was characterized by a drastic decrease in the number of cases, with some small peaks in June 2022 (*n* = 456), September 2022 (*n* = 298), March 2023 (*n* = 150), and April 2023 (*n* = 133). In this period, the number of generated sequences was twelve (12) in June 2022, thirty-two (32) in August 2022, twenty-three (23) in September 2022, fourteen (14) in October 2022, and seventeen (17) in March 2023 (Fig. 4a). In May, June, and July 2022, BA.2 was found in the majority, with some rare detection of BA.1.15.1 (Fig. 3a). We also detected the Delta variant (B.1.617.2) in July 2022 from one patient living in the Kindia region. This patient’s follow-up samples (*n* = 2) were sequenced and gave the same lineage. From August to September, the subvariant BA.5, BA.5.2, BA.5.2.1, BA.5.2.25, and BF.7. were isolated with a predominance of BA.5.2, BA.5.2.1, and BA.5.2.25. The BQ.1.1 was found at the end of September and October from suspect and contact cases and cocirculated with BA.2 and BA.5.2.25. The variant XBB and its derivatives, mostly XBB.1.5.58, were found from January to April. This was also the same trend in the country, as shown in Fig. 4a.

In evolution, we observed that the non-Omicron strains B.1.629, B.1.1.7, R.1, B.1.1.318, B.1.629, and B.1.525 evolved differently and shared a common ancestor with different B.1 or B.1.1 strains. We also observed that Delta, isolated in July 2022, shares a common ancestor with one strain isolated in Guinea. For Omicron, we noted that some lineage BA.1.15 and BA.1.15.1, the BA.5, BQ, and XBB do not share a common ancestor with BA.1 and BA.2. However, they are derivatives from strains that shared a common ancestor with BA.1 and BA.2. This was also observed in XBB group.

**Fig. 5:**
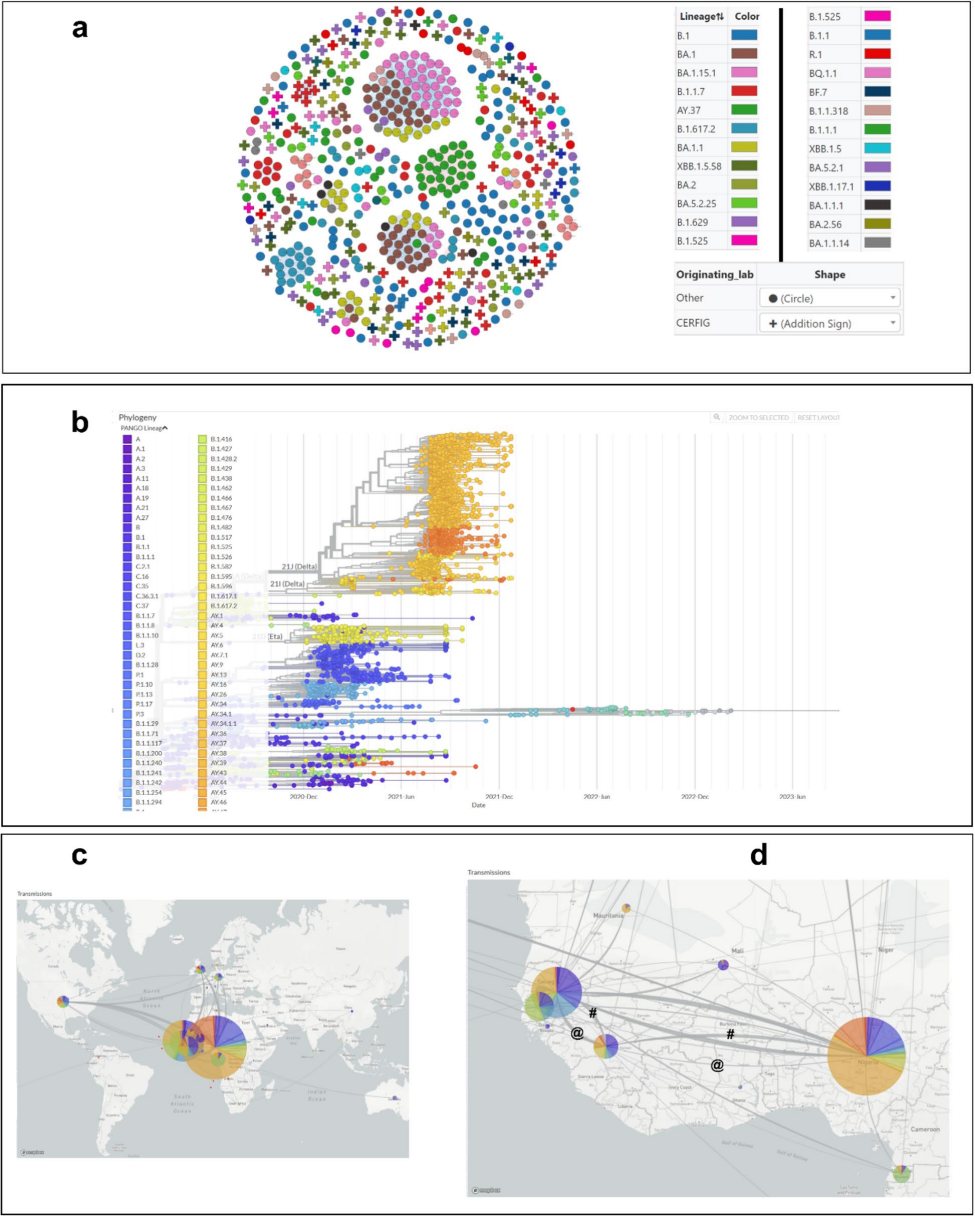
Contact tracing and importation/exportation of *SARS-CoV-2* strains isolated in Guinea. 2D network for different contact tracing in Guinea **a**. Node was filtering prune with the nearest neighbor. Phylogenetic tree of stains isolated in Guinea with most close worldwide strains **b**. The link of a strain isolated from Guinea with the worldwide close strains indicated the treatment of the transmissions map **c** and zoom on Africa with **d**. **# **designated the sequences imported from Senegal and Nigeria, and **@** designated the sequences exported from Guinea to Senegal or Nigeria. The auspice animation can be visualized on https://auspice.us/. The Json file is added in the supplementary data

The contact tracing of different strains isolated in Guinea showed forty-four clusters of connection and 336 singletons (Fig. 5a). In general, we observed strains’ grouping by laboratory and lineage.

Figure 5b-c-d shows the importation/exportation investigation for *SARS-CoV-2* strains isolated in Guinea. The phylogenetic distribution (Fig. 4c-d) shows that some stains were imported into Guinea, and others were exported from the country. The most linked countries were Senegal and Nigeria. Figure 4d shows that the curves of importation and exportation between Senegal and Guinea had the same intensity. For those with Nigeria, the curve of importation from Nigeria is the most intense. For other African countries, we observed another’s connection with Mali, Gambia, Mauritania, Ghana, Equatorial Guinea, and South Africa. The strain exchange was direct or indirect; for European countries, the United Kingdom, Germany, and Denmark were concerned. Here, we also noted the existence of intermediary countries (Senegal or Nigeria). For America, we observed direct and indirect importation or exportation. For the first circulated clade 20 A, importation and exportation were observed with Senegal and an importation from Guinea to Mali and Equatorial Guinea. For 20B strains, exchange between Guinea and Senegal on one hand and Guinea and Nigeria on the other hand were noted. For the 20D clade, importation from Nigeria and importation/exportation with the USA were stressed. For Delta 21 A, clade was imported from Nigeria, and no exportation was noted. For 21 J, exportation from Guinea to Nigeria, but we did not note importation. For BA.1, importation from Nigeria was observed, and exportation to the USA was noted.

## Discussion

Our study contributes to understanding the epidemiological dynamics of *SARS-CoV-2* in Guinea. This study reported the results of almost two years of genomic sequencing. We have detected four variants of concern: Alpha, Delta, Eta, and Omicron. However, most sequences were isolated in 2022, corresponding to the Omicron variant circulation. The Alpha variant was detected in samples collected from January to June 2021 and had a higher prevalence in March 2021. Co-circulation of B.1.1.7 (Alpha) with lineages B.1.1.318 (*n* = 8), R1 (*n* = 7), B.1.525 (*n* = 9), and B.1.629 (*n* = 13) in the period from March to June 2021 was also noted. These results were concordant with the GISAID data from other Guinean laboratories. This period corresponded to the worldwide circulation of the Alpha variant, as reported in different countries like Ghana [29], Mali [30], and Great Britain [31]. In Sierra Leone, co-detection of R1 (80% of cases), B.1.506, and B.1 were notified during the period from December 2020 to February 2021, and the co-detection of B.1.617.2 (Delta), B.1.629, B.1.525 (Eta) and B.1.1.318 from May to August 2021 [32]. Co-circulation of Alpha and Eta variants was notified in Nigeria [33], in Ghana, in addition to the lineage B.1.1.318, in Senegal [34], and in Gambia [35]. For the Delta variant, one of the first detections was from travelers who did not have contact with the local population. In Guinea, the circulating period of Delta corresponds to the period between June to November 2021. This can be confirmed by the sequence’s submission from Guinea on GISAID during that period, in addition to the study of Grayo et al. [36], where there is mention of detected the delta variant in June and July 2021. According to the WHO report by country, there was an increase in the number of COVID-19 cases starting in July 2021 (*n* = 2014) to August when it peaked (*n* = 3740). The Delta lineage circulated in Mali [30] from May to October 2021. A late detection of the Delta variant was noted in July 2022 from a patient in Kindia. An investigation on this patient revealed no travel history, and sequencing of test one and two controls resulted in the same variant. According to Pango-lineage [37], this variant circulated worldwide until December 2022, demonstrating the importance of continuing genomic surveillance activities. For the Omicron variant, nine major subvariants were found with a simultaneous circulation of BA.1, BA.1.15.1, and BA.1.1 in December 2021 and January 2022. The BA.2 variant was only detected from March to July, and then it was cocirculated with BA.5.2 and BA.5.2.1 from August to September. A progressive replacement of BA.1 by BA.2 was observed in Italy from January to April 2022. After that, in June, a co-circulation of BA.2, BA.4 and BA.5 was noted [38]. This study pointed out low BA.4 and BA.5 detection. These subvariants were highly detected in South Africa, the USA, Europe, and Asia [37, 39]. The BQ.1.1 detected were from suspect/contact cases, then unexpectedly in most sequences in October 2022. This indeed demonstrated the impact of genomic surveillance implementation of *SARS-CoV-2* circulating variants in Guinea [39, 40].

Regarding socio-demographics, our results show that there are more males (64%) than females, and the participants aged between 20 and 60 years old were principally affected. In Nigeria, a study [33] reported that the median age of the suspects was 42 years (range 4–86), and the sex distribution was 44% female, 37% male, and 18% unknown. In Brazil, sequenced genomes were from samples collected primarily from females, with a median age of 41.72 years (range: 1 to 90 years of age) [22]. This gives essential information about the population at risk: Guinea’s men and young people, knowing that women cannot be excluded. The results also showed that the most positive PCR COVID-19 (62%) was for control, and for the Omicron circulating period, this category of patients decreased in number. This can be interpreted by the decrease in the COVID-19 panic in the population because the people know more about the disease, and the early detection of cases led to early and adequate treatment during the Omicron period. The suspect/traveler mainly tested positive in the period of Omicron compared to the other period, and the suspect was primarily represented in the positive cases. So, two information can be deducted from these observations. The first is the control politic; even though it existed, it did not detect all cases and was stronger at the beginning of the diseases than at the end. The second is that the virus circulated into the population without contact tracing. Our study also shows that despite the samples collected in different regions of Guinea, most variants were identified with Conakry samples, the economic capital of Guinea. This was consistent with studies performed in Ghana, where the most sequenced samples were isolated in the Greater Accra region [29]. In Burkina Faso, most *SARS-CoV-2* sequences were obtained from samples collected in Bobo Dioulasso and from samples collected at land borders from travelers returning from Côte d’Ivoire and at Bobo Dioulasso airport from a traveler coming from France [40]. This result can be explained by the fact that most African health infrastructures for quick virus characterization are in the capital region. In addition to this limitation, we emphasized virus transport and storage in laboratories in Guinea, which may jeopardize retrospective studies. One solution proposed by *Keita et al.* [41]. was using a rapid diagnostic test cassette as a source of genetic material for genomic surveillance of viruses. So, the laboratories localized in regions without infrastructure can send positive RDT for genomic investigation. This can help to get more data and for the totality of the country. The importation and exportation of the virus during the pandemic showed an exchange between Guinea, Nigeria, and Senegal. These results help to understand the dynamics of diseases and for further investigations to collaborate with those countries for efficient action.

## Conclusion

The implementation of routine sequencing of *SARS-CoV-2* for circulating variant surveillance was a challenge for Guinea laboratories. The results of this study show that regardless of the number of samples sequenced by a laboratory, we can always obtain additional information concerning the virus’s variants that circulate and give public health orientation. So, the more sequences we have, the more the information will be refined. The contact tracing between strains’ isolates in Guinea shows varying clusters, even for isolated lineages. Importation/exportation investigations show links between Guinea and two countries (Senegal and Nigeria). These results show the close relationship between Guinea and these countries and can be used by politics to control infectious diseases. The results showed that the noncontact tracing cases were representative. Implementing national strategies for genomic surveillance of emerging diseases is recommended to coordinate laboratory activities adequately in countries like Guinea. This is also useful for the early detection of variants, understanding the dynamics of the virus, and developing diagnostic and vaccination strategies. The research center can help to assess the implementation, but the national public health laboratories should ensure routine usage. We recommend teamwork between laboratories around the country to collect more analyzable samples for the country, as this will contribute to understanding the virus dynamics. The high implication of authorities and the training of specialists to generate and analyze data constitute another challenge.

## Supplementary Information

Supplementary Material 1.

Supplementary Material 2.

## Data Availability

The author declares that all data in this manuscript are available, and the sequencing data of all samples are submitted in GISAID (gisaid.org) database.

## Abbreviations

COVID-19: Coronavirus Disease 2019
RDT: Rapid diagnostic test
RT-PCR: reverse transcription polymerase chain reaction
SARS-CoV-2: Severe acute respiratory syndrome coronavirus two
VTM: Viral transport medium
RNA: Ribonucleic acid
CERFIG: Centre de Recherche et de Formation en Infectiologie de Guinée
Ct: Cycle threshold
VOCs: variants of concern
WHO: World Health Organization
qPCR: quantitative Polymerase chain reaction
DNA: Deoxyribonucleic acid
GeVarLi: GEnome assembly, Variants calling, and Lineages assignment
GISAID: Global Initiative on Sharing All Influenza Data
GTR: general time reversible
IPG: Institute Pasteur of Guinea
INSP: Institut Nationale de Sante publique
LFHVG: Laboratoire des Fièvres Hémorragique Virales en Guinée
